# Four normative perspectives on public health policy-making and their preferences for bodies of evidence

**DOI:** 10.1186/s12961-020-00614-9

**Published:** 2020-08-24

**Authors:** Casper G. Schoemaker, Jeanne van Loon, Peter W. Achterberg, Frank R. J. den Hertog, Henk Hilderink, Johan Melse, Robert A. A. Vonk, Hans van Oers

**Affiliations:** 1 National Institute for Public Health and the Environment (RIVM), PO Box 1, 3720 BA Bilthoven, The Netherlands; 2 Ministry of Education, Culture and Science, Rijnstraat 50, 2515 XP Den Haag, The Netherlands; 3 The Council for Health and Society, Parnassusplein 5, 2511 VX Den Haag, The Netherlands; 4 Ministry of Health, Welfare and Sport, Parnassusplein 5, 2511 VX Den Haag, The Netherlands

**Keywords:** Evidence-informed policy, Framing, Health policy, Evidence use

## Abstract

Calls for evidence-informed public health policy-making often ignore that there are multiple, and often competing, bodies of potentially relevant evidence to which policy-makers have recourse in identifying policy priorities and taking decisions. In this paper, we illustrate how policy frames may favour the use of specific bodies of evidence. For the sixth Dutch Public Health Status and Foresight report (2014), possible future trends in population health and healthcare expenditure were used as a starting point for a deliberative dialogue with stakeholders to identify and formulate the most important societal challenges for the Dutch health system. Working with these stakeholders, we expanded these societal challenges into four normative perspectives on public health. These perspectives can be regarded as policy frames. In each of the perspectives, a specific body of evidence is favoured and other types of evidence are neglected. Crucial outcomes in one body may be regarded as irrelevant from other perspectives. Consequently, the results of research from a single body of evidence may not be helpful in the policy-making processes because policy-makers need to account for trade-offs between all competing interests and values. To support these policy processes, researchers need to combine qualitative and quantitative methodologies to address different outcomes from the start of their studies. We feel it is time for the research community to re-politicise the idea of evidence use and for policy-makers to demand research that helps them to account for all health-related policy goals. This is a prerequisite for real evidence-informed policy-making.

## Main text

Policy-making processes in public health and healthcare involve accounting for trade-offs between competing interests and values [1–4]. All decisions will have implications for budgets and priorities and are also likely to involve social considerations such as questions of equity, justice or morality [5]. Even the commonly recognised public health goals of improving health and reducing health inequalities can be in tension with one another and deciding which to prioritise is a normative decision [6, 7].

According to Hawkins and Parkhurst [8], the fundamentally political nature of policy-making is often missed by calls for evidence-based public health policy, “*which neglect that there are multiple, and often competing, bodies of potentially relevant evidence to which policy-makers have recourse in identifying policy priorities and taking decisions*”. Interest groups often present their arguments in terms of evidence-based policy, highlighting the bodies of evidence that support the course of action they advocate. What are presented as arguments about evidence are often actually contests between political priorities [8].

Thus far, this distinction was often missed in the literature on evidence-informed public health policy-making [9, 10]. In a literature review, Oliver et al. concluded that most publications still focus on promoting the use of academic research, rather than studying the practices of knowledge generation and implementation in policy-making [11]. Liverani et al. raised similar concerns about “*the public health community’s tendency to depoliticize the idea of evidence use, evaluating policy making processes simply by whether, how much, or how quickly pieces of evidence are taken up by policy makers*” [5]. In their systematic review Liverani et al. found very few publications that applied policy science perspectives to understand the use of evidence in policy-making [5]. As a result, it remains unclear how the political characteristics of a given public health issue might determine the use of a specific body of evidence, while other evidence is neglected.

The policy science literature on ‘frames’ and ‘framing’ may be useful to shed light on this issue [12–14]. Frames are useful concepts in understanding the nature of political debates by providing an explanation of structure, agency and instruments used in the policy process [1, 15, 16]. “*Both overtly and covertly, frames highlight certain aspects of a problematic situation, while obscuring others in order to define problems, diagnose causes, make moral judgements and suggest remedies*” [15]. Framing assumes a strategic use of evidence in policy-making [17]. Research is best seen as helping policy-makers decide which policies are best suited to the realisation of their ideologies and interests [18, 19]. According to Hoppe, in an ‘adversarial model’ of evidence use in policy-making, the struggle between group interests may function as “*selection environment for scientific arguments that underpin political positions and decisions*”. Every interest group will look for the specific body of evidence that substantiates its own political standpoint [20]. In other words, political diversity coincides with epistemological and methodological diversity [21].

In this paper, we will illustrate how policy frames in public health may favour the use of specific bodies of evidence. This is based on the study of the sixth Dutch Public Health Status and Foresight (PHSF 2014) report [22], in which future trends in population health and healthcare expenditure were used to formulate the most important societal challenges with stakeholders. In a deliberative dialogue, these societal challenges were expanded into four normative perspectives on public health. Each perspective centres on one of the societal challenges, with the other challenges subordinated [23, 24]. In other words, they serve as policy frames [15, 25]. To identify potential interrelationships between the perspectives, we organised 4 expert sessions (on life expectancy and burden of disease, participation and exclusion, autonomy of civilians and patients, and health budget and economy) with more than 50 experts to explore how engagement based on each particular perspective would affect all societal challenges [22]. This approach clarified areas in which positive spin-offs could occur and win–win strategies could be created (opportunities). To give an example, promoting health may improve participation in vulnerable social groups and, as a result, the overall burden of disease could lighten [22]. The PHSF 2014 report also identified areas in which negative side effects could arise and where political choices would be necessary (options or dilemmas). For instance, if more room is created for diversity and freedom of choice, there will be some vulnerable groups that are insufficiently equipped to cope with it [22].

In this paper, we will add an epistemological and methodological dimension to this discussion about policy-making in public health [21]. In each of the perspectives, a specific body of evidence is favoured and other types of evidence are neglected. Crucial outcomes in one body may be regarded as less important or even irrelevant from other perspectives. Consequently, the results of research from a single body of evidence may not be helpful in the policy-making processes because policy-makers need to account for trade-offs between all competing interests and values. To support these policy processes, researchers need to combine qualitative and quantitative methodologies to address different outcomes in the design of their studies [21]; the implications for evidence-informed health policy-making are discussed.

### Framing public health in four perspectives

The trend scenario of the PHSF 2014 was based on analysis of historical trends and on a combination of demographic and epidemiological projections (assuming ‘business-as-usual’). Ageing was found to be a key factor in the trend scenario of the PHSF 2014 [22]. By 2030, Dutch life expectancy would increase by a further 2–3 years. As a result, the percentage of people living with chronic illnesses, including dementia, would keep rising to 40% in 2030. The difference in life expectancy between people with low and high levels of education would remain of 6 years or grow slightly. Some negative trends in lifestyle factors – smoking and overweight – have been mitigated, but it remains to be seen whether that will be sustained. One of the most uncertain trends was the future evolution and impacts of healthcare expenditure [22].

These major trends in public health and healthcare in The Netherlands served as a starting point for a deliberative dialogue in three meetings with more than 100 stakeholders from a broad range of sectors (health professional, patient organisations, unions, students, insurance companies, national and local health policy-makers) [22]. Four societal challenges for public health and healthcare were identified and formulated, as follows: (1) to keep people healthy as long as possible and cure illness promptly; (2) to support vulnerable people and enable social participation’ (3) to promote individual autonomy and freedom of choice; and (4) to keep healthcare affordable.

Working with stakeholders, we framed these societal challenges into four perspectives on public health. These are entitled In the Best of Health (IBH), Everyone Participates (EP), Taking Personal Control (TPC) and Healthy Prosperity (HP) (Table 1). A survey in the Dutch adult population showed all four perspectives to be recognisable and sufficiently distinctive [22].

**Table 1:**
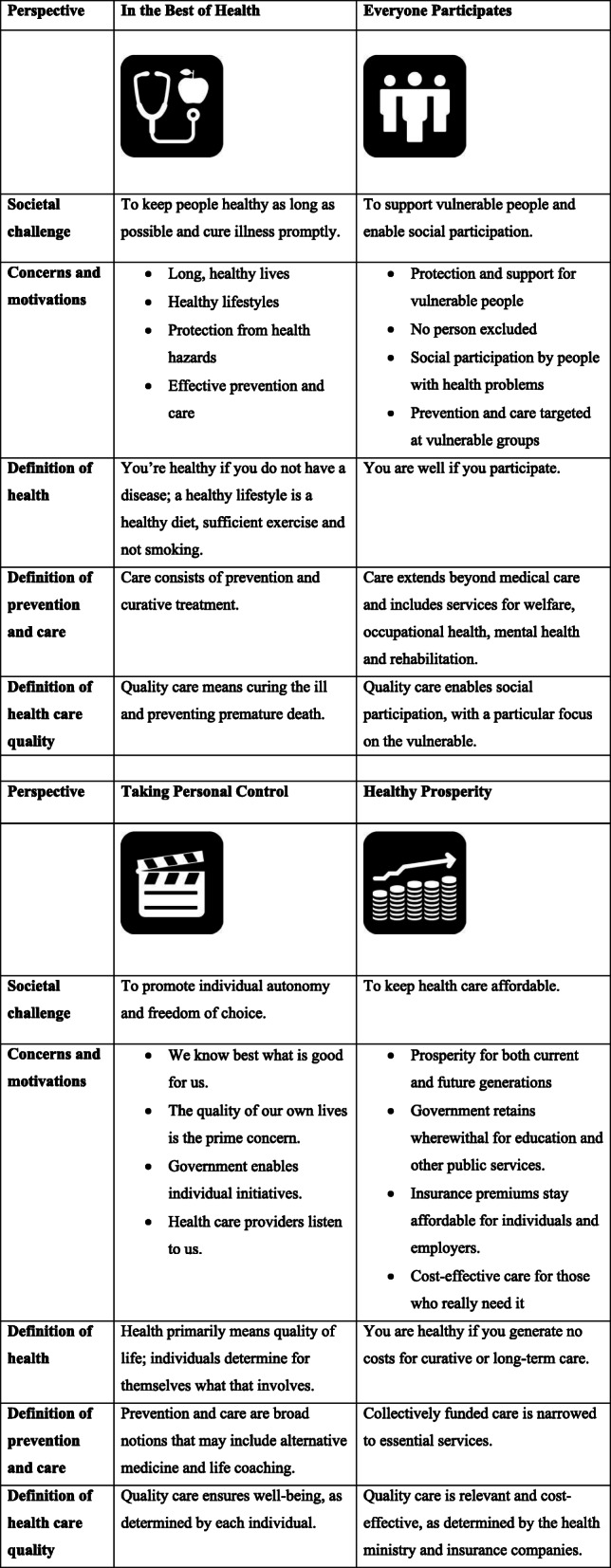
Four normative perspectives on public health: societal challenges, concerns and motivations, definitions of health, prevention, care and quality of care

As can be seen in Table 1, notions such as ‘health’, ‘prevention’, ‘healthcare’ and ‘quality of care’ have different meanings in each perspective. According to the IBH perspective, ‘health’ is understood mainly as the absence of disease. By contrast, in EP, clinical diagnoses are less relevant, since social participation is the vital concern. The third perspective, TPC, contains no universally valid conception of health; individuals determine that for themselves. In the fourth perspective, HP, ‘health’ stands mainly for as little healthcare spending as possible [22].

Furthermore, the interpretations given to the notion of ‘quality of care’ are different in each perspective [26, 27]. Under IBH, healthcare quality means that illnesses are cured and premature death is avoided. Under EP, the emphasis is on the effects of healthcare on social participation of the disadvantaged. In TPC, each individual determines what good quality care is and, in HP, good care is primarily cost-effective care for those who really need it [22].

### How each perspective favours the use of a body of evidence

These four normative perspectives have epistemological and methodological dimensions as well – in each of the perspectives, a specific body of evidence is favoured. As can be seen in Table 2, under IBH, meta-analyses of randomised controlled trials (RCT) are thought to be the best evidence whereas, in the EP perspective, population statistics, quasi-experiments and action research are the preferred research methods. In TPC, qualitative research is preferred. In HP, quantitative economic analyses, and especially social cost-benefit analysis, is warranted.

**Table 2:**
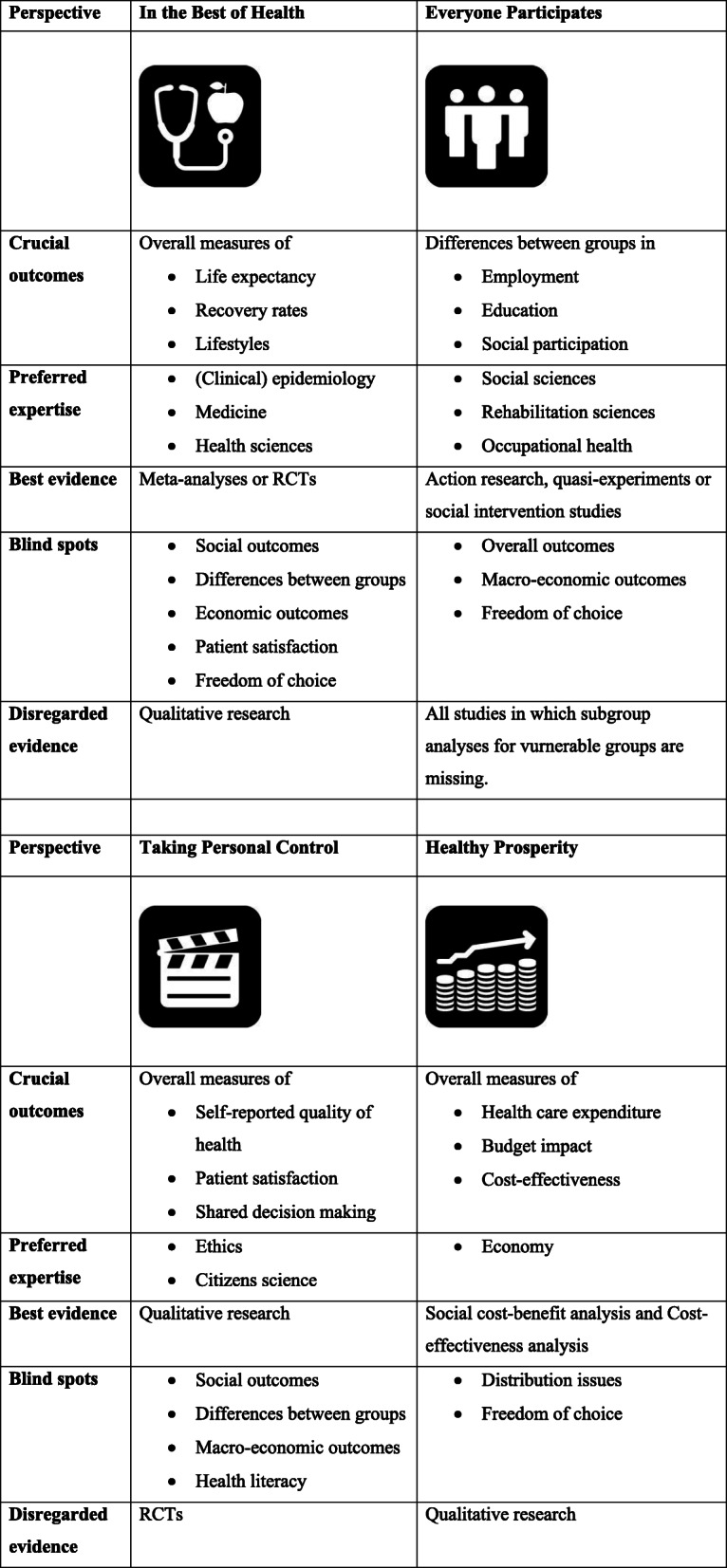
Four normative perspectives on public health: crucial outcomes, preferred expertise, evidence, blind spots and disregarded evidence

In each perspective, different outcomes are regarded relevant. According to the IBH perspective, mean overall population measures of life expectancy, disease prevalence, recovery rates and lifestyles are crucial outcomes. Under EP, the emphasis is on differences between population groups in disabilities, employment, education and societal inclusion. In TPC, individual self-reported quality of health is the main outcome and, in HP, overall budget impact and economic growth is what matters most as an outcome.

Interestingly, the outcomes relevant to three of the perspectives have been captured in the well-known Triple Aim framework for quality improvement in healthcare [26]. In a recent review, Mery et al. proposed to include equity on a population level (a goal in the missing perspective EP) as an additional fourth aim [27].

Crucial outcomes and preferred methodologies in one perspective may be blind spots in the evidence base for other perspectives. In the hierarchy of evidence under IBH, qualitative research is ranked very low, while in TPC this is the preferred methodology and RCTs are under suspicion and disregarded. Moreover, in the welfare theory underlying the social cost-benefit analyses in HP, socio-economic differences are non-essential [28]. In these analyses, taxes aimed at levelling the differences – preferred policies in EP – will always jeopardise economic growth.

Obviously, some bodies of evidence may be combined with greater ease in mixed-methods research while, for other combinations, researchers need to make additional efforts. IBH had been the leading perspective for the development of clinical treatment guidelines, with an emphasis on RCTs [10, 17]. To take resource use into account (HP) is not problematic – nowadays, it is usance to add an economic evaluation to new RCTs. Thus far, evidence on equity and social outcomes in terms of employment or social participation (EP) are seldom addressed in clinical guidelines [29]. Recently, a conceptual framework was described to consider health equity in the Grading Recommendations Assessment and Development Evidence (GRADE) guideline development process (EP) [30]. The authors proposed five methods for explicitly assessing health equity in guidelines, namely (1) include health equity as an outcome; (2) consider patient-important outcomes relevant to health equity; (3) assess differences in the relative effect size of the treatment; (4) assess differences in baseline risk and the differing impacts on absolute effects; and (5) assess the indirectness of evidence to disadvantaged populations and/or settings.

The emphasis on experimental trials in the hierarchy of evidence under IBH did forestall the use of qualitative research in guidelines [10, 17]. However, the GRADE Working Group acknowledged that, for guideline panels, “*the relative importance given to outcomes should reflect the perspective of those who are affected. When the target audiences for a guideline are clinicians and the patients they treat, the perspective would generally be that of the patient*” [31]. In other words, to judge the relevance of outcomes in quantitative RCTs, qualitative methods, e.g. thematic syntheses and focus groups, are clearly needed to address the perspective of those affected. Obviously, not all tensions can be solved. For most guideline makers, there remains a clear tension between their emphasis on the results of experimental trials and the freedom for patients and clinicians not to follow the guideline as a result of shared decision-making (TPC) [32].

## Discussion

According to Parkhurst, most past work on the use of evidence in policy-making “*has assumed that more evidence use is inherently better evidence use. Such a belief appears to rest on an assumption that evidence works to serve a problem-solving role where all outcomes have been agreed. But such situations are the exception, rather than the rule, in policy making. As such, many evidence utilisation concepts and strategies arising from this position have typically been under-specific – failing to ask which evidence for what goals in particular*” [17].

The PHSF 2014 perspectives illustrate how political diversity coincides with epistemological and methodological diversity [21, 33]. Firstly, these perspectives serve as policy frames [15, 25], highlighting certain aspects of a problematic situation, while obscuring others in order to define problems, diagnose causes, make moral judgements and suggest remedies. Secondly, in each of the perspectives, a specific body of evidence is favoured, and other types of evidence are neglected. Crucial outcomes in one body may be regarded as irrelevant in another body of evidence. Consequently, the results of research from one single body of evidence may not be helpful in the policy-making processes in public health and healthcare because (unintentionally) some of the competing interests and values are highlighted while others are neglected [1, 2, 7, 19, 34].

We see the relation between research and policy as a two-way negotiation in which both partners learn from each other [11]. Researchers need to learn that policy-making is a complex, non-linear process driven by multiple elements of which research knowledge is only one. Other elements include organisational structures, media, public opinion and budgets. Policy-makers need to understand how to request the evidence that really informs their decisions.

In health policy-making, there is clearly a need for evidence from more than one body of evidence, to inform the policy-makers, on several relevant policy goals and priorities [35, 36]. In hindsight, IBH and to a lesser extent EP have long been the leading perspectives in public health in the Netherlands, including the PHSF reports. This may explain the tension between a medical, epidemiological approach guided by the national PHSF reports and a more societal frame of the policy-makers [37].

We feel it is time for the health research community to re-politicise the idea of evidence use in policy-making [4, 5, 7]. One of the reasons why policy-makers may not use research in policy-making may be the fact that much of the available research comes from single bodies of evidence [17]. In other words, methodological choices, inherent to a body of evidence, may unintentionally jeopardise the usability of the results in health policy-making. If researchers really want their work to inform the complex policy-making process, in which policy-makers evaluate competing social outcomes and value systems and make political decisions, researchers should recognise a range of different types of methodologies and outcomes as relevant and combine them, from the start, in the design of mixed-methods research [17, 21, 31].

## Conclusions

In the PHSF 2014 report, we highlighted a number of different opportunities for policy-makers to establish links between the perspectives and their respective challenges [22]. In this paper, we added an epistemological and methodological dimension to this discussion [21]. Policy-makers need to account for trade-offs between all competing interests and values. To support these policy processes, research from a single body of evidence will not suffice [17]. Researchers need to combine qualitative and quantitative methodologies to address different outcomes from the start of their studies. Some bodies of evidence can be combined in a mixed-methods research design with great ease while, for other combinations, additional efforts need to be made. For this kind of research that takes interests and values into account, stakeholder involvement is clearly a requisite [36, 38]. We hope this approach helps researchers and policy-makers to recognise synergies and dilemmas between different bodies of evidence. Finally, this approach may be helpful to find win–win strategies in conjunction with other domains outside public health, e.g. climate change, transport, or migration, and between domains [39].

## Data Availability

The datasets used and/or analysed during the current study are available from the corresponding author on reasonable request.

## Abbreviations

EP: Everyone Participates
GRADE: Grading Recommendations Assessment and Development Evidence
HP: Healthy Prosperity
IBH: In the Best of Health
PHSF: Public Health Status and Foresight
RCT: randomised controlled trial
TPC: Taking Personal Control
